# Enhanced response of melanoma cells to MEK inhibitors following unbiased IGF-1R down-regulation

**DOI:** 10.18632/oncotarget.19286

**Published:** 2017-07-17

**Authors:** Naida Suleymanova, Caitrin Crudden, Claire Worrall, Anica Dricu, Ada Girnita, Leonard Girnita

**Affiliations:** ^1^ Department of Oncology and Pathology, Cancer Center Karolinska, Karolinska Institutet, Karolinska University Hospital, Stockholm, Sweden; ^2^ Biochemistry Unit, University of Medicine and Pharmacy of Craiova, Craiova, Romania; ^3^ Dermatology Department, Karolinska University Hospital, Stockholm, Sweden

**Keywords:** biased signaling, rtks, functional selectivity, cancer, targeted therapy

## Abstract

Due to its ability to compensate for signals lost following therapeutic MAPK-inhibition, insulin-like growth factor type 1 receptor (IGF-1R) co-targeting is a rational approach for melanoma treatment. However IGF-1R conformational changes associated with its inhibition can preferentially activate MAPK-pathway in a kinase-independent manner, through a process known as biased signaling. We explored the impact of biased IGF-1R signaling, on response to MAPK inhibition in a panel of skin melanoma cell lines with differing MAPK and p53 mutation statuses. Specific siRNA towards IGF-1R down-regulates the receptor and all its signaling in a balanced manner, whilst IGF-1R targeting by small molecule Nutlin-3 parallels receptor degradation with a transient biased pERK1/2 activity, with both strategies synergizing with MEK1/2 inhibition. On the other hand, IGF-1R down-regulation by a targeted antibody (Figitumumab) induces a biased receptor conformation, preserved even when the receptor is exposed to the balanced natural ligand IGF-1. This process sustains MAPK activity and competes with the MEK1/2 inhibition. Our results indicate that IGF-1R down-regulation offers an approach to increase the sensitivity of melanoma cells to MAPK inhibition, and highlights that controlling biased signaling could provide greater specificity and precision required for multi-hit therapy.

## INTRODUCTION

Malignant melanoma is the deadliest form of skin cancer and has shown increasing incidence since the 1970s. Although surgical intervention is curative at early stages, melanoma undergoes rapid dissemination, at which point it is often fatal, with a 5 year survival rate below 20% [1]. The last few decades have seen significant advancement in our understanding of the melanoma pathogenesis, in particular recognition of the hyperactive mitogen activated protein kinase (MAPK) signaling cascade, most frequently through oncogenic mutation in the B-RAF or RAS genes, as a key molecular mechanism driving the disease [2]. This aberrant MAPK pathway mediates a spectrum of cancer promoting bioactivities, including survival, proliferation and metastasis [3, 4]. While the development of targeted MAPK therapeutics gained momentum and held a lot of promise, they turned out to be associated with rapid resistance [5–8]. Mechanisms of resistance differ but generally encompass signal re-routing to allow a subset of cells to adapt by enriching alternative pathways. In this context, MAPK inhibition has been shown to reduce proteolytic removal of multiple receptor tyrosine kinases (RTKs), directly mediating resistance by increasing cell surface receptor levels [9]. RTK rich sub-clones overcome the MAPK-inhibitory drugs by being able to utilize alternative signaling pathways for survival, proliferation and most critically - metastasis. Various co-targeting approaches combining inhibition of MAPK and RTKs are being investigated [10, 11] and *in vivo* studies show promising combination results [12]. Among various RTKs found to be associated with resistance to MAPK [13–15], studies on post-relapse tumor samples have shown increased expression and/or signaling of the insulin-like growth factor type 1 receptor (IGF-1R) [14, 16, 17].

The IGF-1R is a highly cancer relevant RTK, explored extensively in anti-cancer therapeutic approaches [18–21], all aiming to inhibit receptor kinase activity either by preventing ligand–receptor interaction (e.g. blocking antibodies) or mitigating the effects of this interaction (e.g. tyrosine kinase inhibitors (TKIs)). While the main purpose - limiting receptor kinase activity - is achieved in all of these strategies, some intriguing results revealed an unexpected dissociation of receptor trafficking from its kinase activity, as IGF-1R inhibition could also lead to its down-regulation [18-20, 22-26]. Consequently, the receptor conformation associated with down-regulation was demonstrated to initiate kinase-independent, β-arrestin-mediated signaling, mostly through the MAPK pathway [18-20, 27]. This ability of a receptor to preferentially activate only a certain subset of signaling mechanisms triggered by the natural, balanced ligand (IGF-1) is termed biased signaling or functional selectivity [18]. In analogy with the case of the larger GPCR family, agonists capable of selectively activating downstream signaling are defined as biased agonists [28–32].

Recently we demonstrated such a paradigm for the IGF-1R targeting antibody Figitumumab (CP-751871, herein referred to as CP) [25]. Instead of completely inactivating the system, CP acts as a biased agonist, by inducing a partially active receptor conformation that activates a sustained, β-arrestin-dependent MAPK cascade, limiting its inhibitory effect [33, 34]. A more recently described IGF-1R down-regulation strategy involving the small molecule Nutlin-3 acts through redistribution of the E3 ligase Mdm2, away from p53 and towards the IGF-1R [35]. This setting also leads to a partially active receptor conformation that preferentially activates ERK1/2, although this type of biased signaling is transient in nature and thus differs from that induced by CP.

The corollary of these studies is that β-arrestin-biased signaling plays a significant role in determining the overall effects of IGF-1R mono-targeting approaches [18-20, 25, 35]. There is evidence to suggest that co-targeting IGF-1R could enhance melanoma response to MEK inhibitors [14, 17, 36–40], but the role of β-arrestin-biased signaling in dual targeting systems is not known. Thus, the aim of this study was to investigate the potential of balanced versus stable/transient biased IGF-1R down-regulation to enhance the response to MAPK inhibition in melanoma.

## RESULTS

### Effects of MEK1/2 inhibition on RAS/BRAF mutant melanoma cells

In normal cells the RAS/RAF/MEK/ERK pathway is triggered by a plethora of external stimuli such as adhesion molecules, cytokines and growth factors. In some cancer cells, including melanoma, this pathway is hyperactive due to oncogenic mutation of upstream hubs. Therefore, we initially aimed to characterize our experimental model regarding the status of MAPK activation, as well as sensitivity to the prototypic MEK1/2 inhibitor U0126. We used a panel of melanoma cell lines with a range of RAS/RAF mutation and p53 status: DFB contains an activating BRAF mutation and wild type p53, Mel28 contains a BRAF mutation with mutated p53, and BE contains an NRAS mutation and a hot-spot mutation in p53 [41].

**Figure 1:**
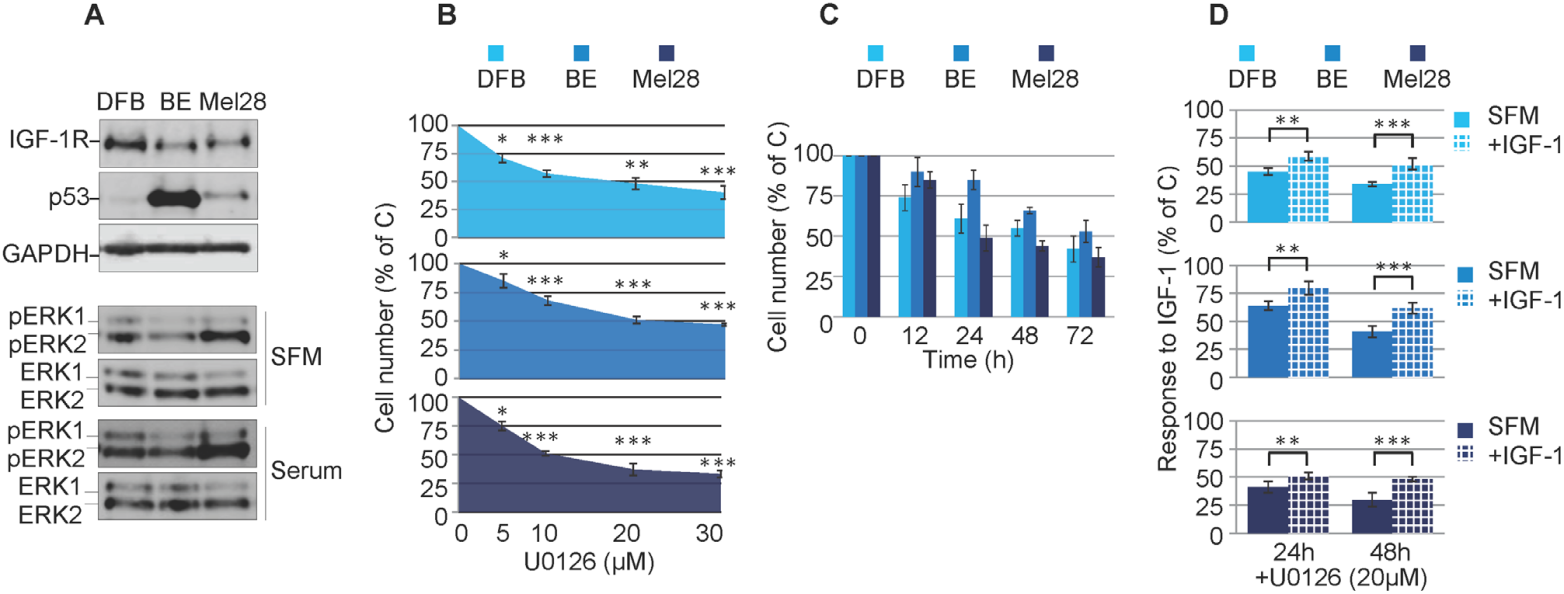
Effects of MEK1/2 inhibition on RAS/BRAF mutant melanoma cells **(A)** Cells grown in complete media (10% FBS) were lysed and analyzed by Western Blot (WB) for IGF-1R, p53 and phosphorylated ERK1/2 (pERK1/2) alongside GAPDH or total ERK1/2 (ERK1/2) as loading controls. **(B, C, D)** Effect of MEK1/2 inhibitor U0126 on melanoma cell viability. DFB, BE and Mel28 cells were treated as indicated, in serum (B, C) or SFM (D), total cell number was evaluated by PrestoBlue fluorescence, and expressed as a % of DMSO (solvent only) treated control. Data displayed as mean ± S.E.M from three independent experiments. (B) Dose response, with increasing doses of U0126 for 72 h (C) Time response, 20 μM U0126 for times indicated. (D) Cells in serum free media (SFM) or 50 ng/mL IGF-1 were treated as indicated. Statistical analysis: (B) U0126 treated cells compared with control-treated cells. (D) Cells treated with U0126 in the presence of IGF-1 compared to SFM conditions. *P<0.05, **P<0.01, ***P<0.001.

Initial characterization by western blot analysis (WB) of the cell panel demonstrated high basal p53 levels in the p53 mutant BE and Mel28 cells and low levels in the p53 wild type cells DFB (Figure 1A). On the other hand the levels of ERK1/2 activation in cells cultured in serum free media (SFM) were not associated with the RAS/BRAF mutation (Figure 1A). For instance Mel28 displayed higher levels of pERK1/2 than DFB, despite the fact that both cell lines harbor the same ^V600E^BRAF mutation. The ^Q61R^NRAS mutant BE cells exhibited only moderate levels of ERK1/2 activation, slightly lower than DFB. Furthermore, the levels of ERK1/2 activation were increased by culturing the cells in serum conditions indicating that maximum MAPK activation is not reached solely by oncogenic mutations within the MAPK pathway (Figure 1A). Finally we investigated the sensitivity to a MEK1/2 inhibitor by measuring the cell viability of melanoma cell lines following U0126 treatment. 72 h of treatment with MEK1/2 inhibitor decreases the cell viability of all melanoma cells in a dose-dependent manner, with the ^V600E^BRAF positive cells (Mel28 and DFB) being slightly more sensitive, each with an IC50 of around 20 μM (Figure 1B).

Based on these experiments, a dose of 20 μM U0126 was chosen to evaluate response over time. All tested cell lines treated with MEK1/2 inhibitor demonstrated a time-dependent response in cell viability. Of note, in both the dose and time response experiments, a proportion of 30-40% of the cells were still alive even at the highest tested doses, and longest times (Figure 1B, 1C). Given that these experiments were performed in the presence of serum-containing media, this does not allow us to assess the potential involvement of survival factors normally present in serum that could compensate for the MAPK inhibition. For this reason and to specifically measure the possible IGF-1R-contribution in mediating protective effects, we performed the cell viability experiments in serum free media supplemented only with IGF-1 (Figure 1D). In the absence of serum, U0126 further decreases the cell viability by 20-30% when compared with the same dose in the presence of serum, whereas the addition of IGF-1 has protective effects and almost completely rescued the cells up to the levels observed in serum (Figure 1D).

Taken together, the proliferation data revealed that MEK1/2 inhibition decreases the total number of cells in all melanoma cell lines. This was enhanced in the absence of serum, whereas re-addition of only IGF-1 almost completely compensated in protecting melanoma cells against MEK1/2 inhibition by U0126.

### Characterisation of effect of balanced versus biased targeting strategies on IGF-1R expression

Having shown that IGF-1R signalling limits the effects of MEK1/2 inhibition in melanoma cells, we next evaluated different approaches to prevent IGF-1R activation. We tested three strategies that down-regulate the IGF-1R through different mechanisms with parallel balanced or biased signaling activation. Firstly, we used small interfering RNA (siRNA) against IGF-1R to block IGF-1R synthesis at the level of mRNA [35, 42] without modifying receptor downstream signaling (i.e. balanced down-regulation). Secondly, IGF-1R targeting antibodies (e.g. CP) that bind the receptor, preventing its interaction with the ligand, but at the same time modify the receptor conformation to trigger down-regulation and selectively sustain MAPK activation (biased agonist down-regulation) [25]. And lastly, Nutlin-3, recently identified to down-regulate IGF-1R by causing accumulation of Mdm2 - a major ubiquitin ligase for the IGF-1R, causes transient MAPK activation (biased agonist down-regulation) [35].

**Figure 2:**
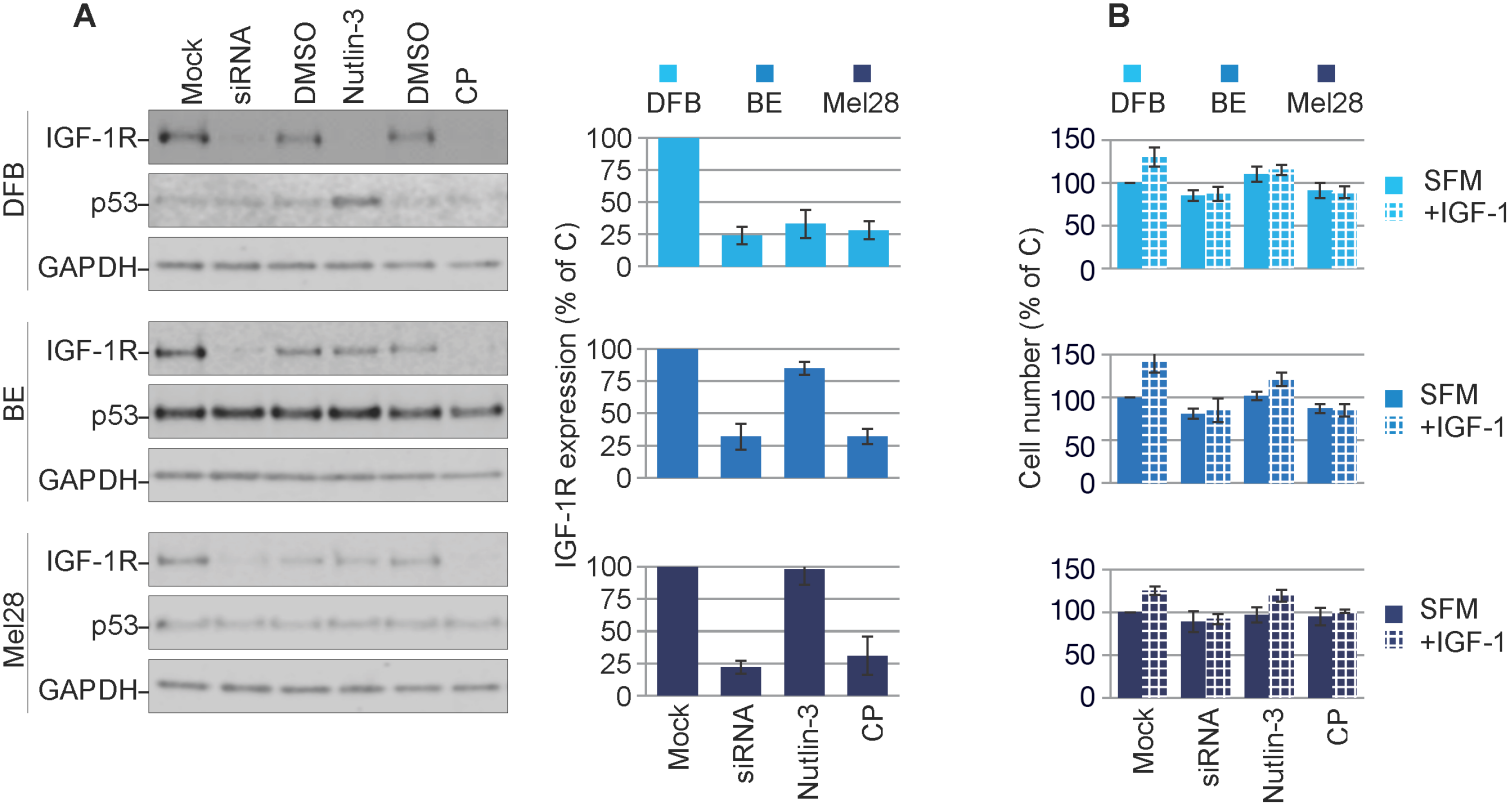
Characterization of balanced versus biased targeting strategies on IGF-1R expression **(A)** DFB, BE and Mel28 melanoma cells were either transfected with siRNA towards IGF-1R (48 h) or treated with 1 μM Nutlin-3 (12 h) or 100 ng/mL CP (12 h), alongside non-target siRNA-transfected or solvent only controls (Mock/DMSO). Lysates were analyzed by WB for total IGF-1R, p53, and GAPDH as a loading control. IGF-1R signals were quantified by densitometry, normalized to GAPDH and expressed as a % of the IGF-1R in the control-treated cells (graphs). Data correspond to the mean ± S.E.M. from three independent experiments. **(B)** Parallel samples treated as in (A) were serum starved and stimulated or not with IGF-1 (50 ng/mL) for 24 h. Total cell number was assayed by PrestoBlue fluorescence, and expressed as % of control (Mock, SFM) treated cells. Data indicates mean ± S.E.M from three independent experiments. Statistical analysis: Cells treated in the presence of IGF-1 compared to SFM conditions. *P<0.05, **P<0.01, ***P<0.001.

In the next experiments we verified the effects of treatment with IGF-1R siRNA [35], anti-IGF-1R antibodies or Nutlin-3 [25, 35] on p53 and IGF-1R expression. In DFB all three approaches decrease the IGF-1R to a similar extent (70 - 80%). In BE and Mel28, the siRNA and CP effect of lowering receptor level are consistently strong, like in DFB, but Nutlin-3-dependent removal of the IGF-1R in BE is limited to about 25% and notably does not occur at all in Mel28. This data is in line with previously reported data demonstrating a requirement of Mdm2 release from p53 for IGF-1R down-regulation (BE has a lower Mdm2 level, whereas in Mel28 there is no Nutlin-3-induced changes in Mdm2 levels) [35]. Additionally, in wild type p53 DFB cells, p53 accumulates in response to Nutlin-3 treatment, whereas no noteworthy changes in p53 were observed following siRNA or CP treatment, or in any of the other cell lines regardless of treatment regimen (Figure 2A). The levels of IGF-1R following different treatments were confirmed by densitometry quantification of multiple experiments (Figure 2A, graphs).

To functionally verify the IGF-1R down-regulation we measured the proliferative response to IGF-1 stimulation following treatment (Figure 2B). Following IGF-1R down-regulation by either siRNA or CP alone, all cells became unresponsive to IGF-1 stimulation. In the case of Nutlin-3 treatment, only the DFB cells and to a much lesser extent the BE cells, demonstrated an impaired response to IGF-1, which parallels the decreased levels of the IGF-1R. Mel28 cells were unresponsive to Nutlin-3 treatment, both in terms of IGF-1R reduction and response to IGF-1.

These results confirm that all three strategies act to down-regulate the IGF-1R and that decreased receptor level is associated with less proliferative response to ligand stimulation.

### Characterisation of balanced versus biased targeting strategies on IGF-1R signaling

**Figure 3:**
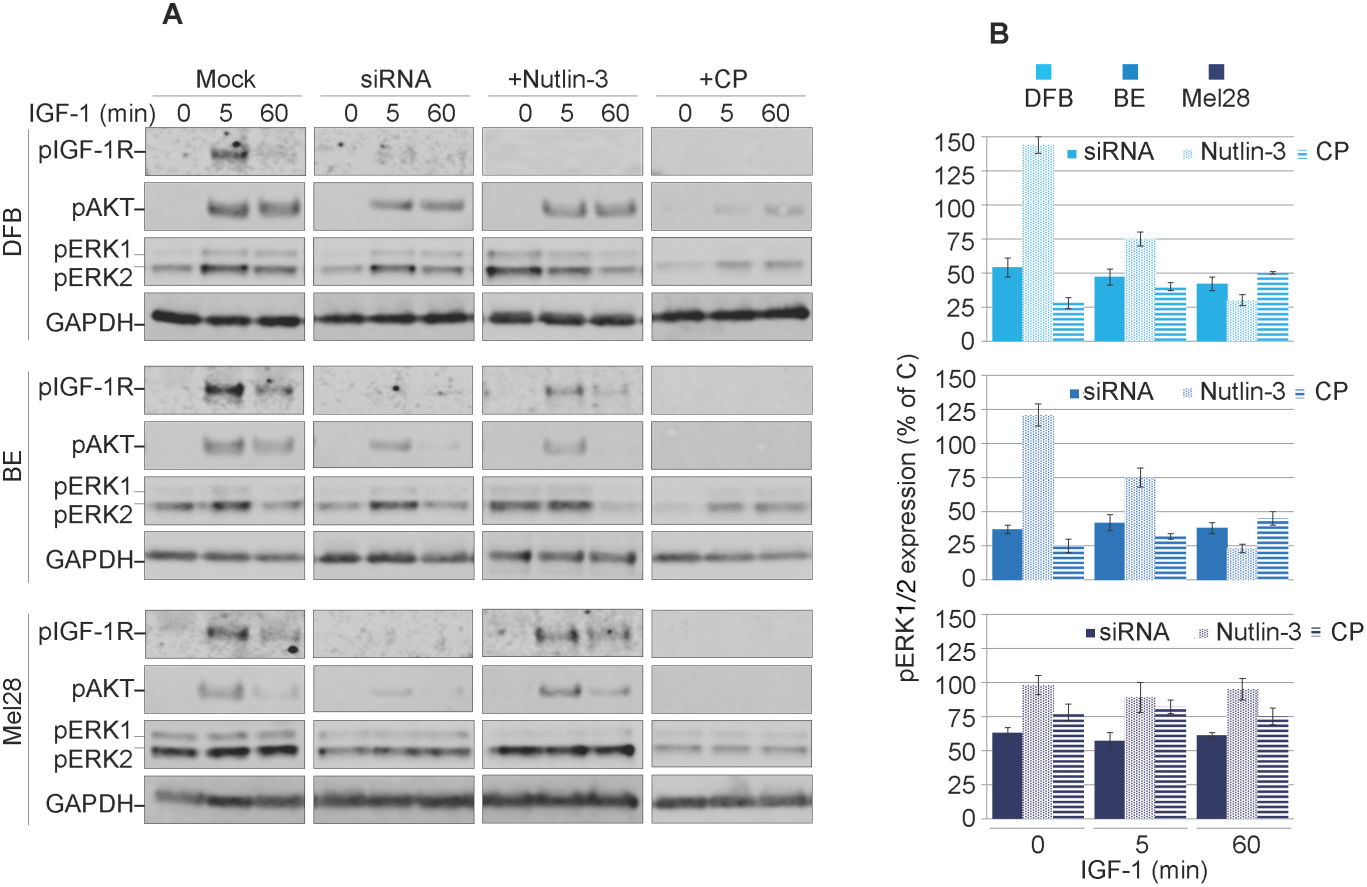
Characterization of balanced versus biased targeting strategies on IGF-1R signalling **(A)** DFB, BE and Mel28 melanoma cells were either transfected with siRNA towards IGF-1R (48 h) or treated with 1 μM Nutlin-3 (24 h) or 100 ng/mL CP (24 h), alongside mock controls. Serum starved cells were stimulated with IGF-1 (50 ng/mL) for 0, 5 and 60 min. Cell lysates were analyzed by WB for phosphorylated (p) versions of IGF-1R, Akt and ERK1/2, alongside GAPDH as a loading control. **(B)** pERK1/2 signals were quantified by densitometry, normalized to total ERK1/2 and expressed as a % of pERK1/2 in the mock-treated cells for each time point. Data correspond to the mean ± S.E.M. from three independent experiments.

Previous reports demonstrated that the IGF-1R conformational change induced by various targeting agents could trigger transient or sustained biased downstream MAPK signaling [25, 35]. Therefore, in the next experiments we explored the possible agonistic properties of CP, Nutlin-3 and IGF-1R siRNA. To reveal the effects of biased signaling associated with conformational changes of the IGF-1R, we initiated the receptor down-regulation by treating the cells with CP, Nutlin-3 or siRNA in the presence of the ligand (10% serum). The cells were then serum starved and stimulated with IGF-1 for up to 60 min. The subsequent tyrosine kinase activation of the receptor and the two main downstream signaling pathways: IRS/PI3K/Akt and RAS/RAF/MEK/ERK, were analyzed by WB detection of phosphorylated forms of IGF-1R, Akt and ERK1/2 (Figure 3A). In untreated cells, upon ligand stimulation, the IGF-1R kinase activity clearly increases after 5 min of IGF-1 stimulation, as demonstrated by its enhanced phosphorylation levels. Subsequently, both main downstream signaling pathways were activated as demonstrated by ERK1/2 and Akt phosphorylation (Figure 3A).

Following treatment with either siRNA, Nutlin-3 or CP, phosphorylation of the IGF-1R (indicating its kinase activity) is greatly impaired, confirming that the receptor is indeed removed from the cell surface and not available for ligand binding (Figure 3A). These effects were observed in all cell lines with the exception of Mel28 treated with Nutlin-3, which preserved functional IGF-1R. The levels of Akt phosphorylation decreased alongside pIGF-1R, indicating the need for a kinase-competent IGF-1R for PI3K/Akt activation. On the other hand, the levels of MAPK activity as measured by ERK1/2 phosphorylation critically diverge dependent upon treatment regimen, revealing an intriguing pattern for functional selectivity of the signaling as confirmed by signal quantification from multiple experiments (Figure 3B). In the case of siRNA treatment, the levels of pERK1/2 in response to IGF-1 stimulation were decreased by about 50-60% at all investigated time points, indicating a balanced dampening effect on MAPK activity, with proportionally decreased IGF-1R and Akt signaling. On the other hand, following Nutlin-3-induced IGF-1R down-regulation the levels of ERK1/2 phosphorylation in DFB and BE cell lines, demonstrated a decreasing trend over time following ligand stimulation, revealing an early but short-lived biased MAPK signaling. Notably this did not occur in Mel28, although pERK1/2 was higher before stimulation (time 0). Conversely, in cells with CP-induced IGF-1R down-regulation, IGF-1 stimulation induced a sustained MAPK-biased signaling: although the levels of pERK1/2 were generally lower as compared with control cells at all times, the signals remained increased even at 60 min following IGF-1 stimulation. It should be noted here that this biased signaling is induced in response to the prototypical balanced ligand (IGF-1), indicating that a biased-receptor conformation stabilized by CP-pretreatment is preserved even after CP treatment is removed.

Taken together these results demonstrate decreased IGF-1R signaling following all treatment regimens, balanced in the case of siRNA, and transient or sustained biased MAPK signaling in the case of Nutlin-3 and CP, respectively.

### Effects of balanced versus biased IGF-1R down-regulation on melanoma response to MEK1/2 inhibitor

**Figure 4:**
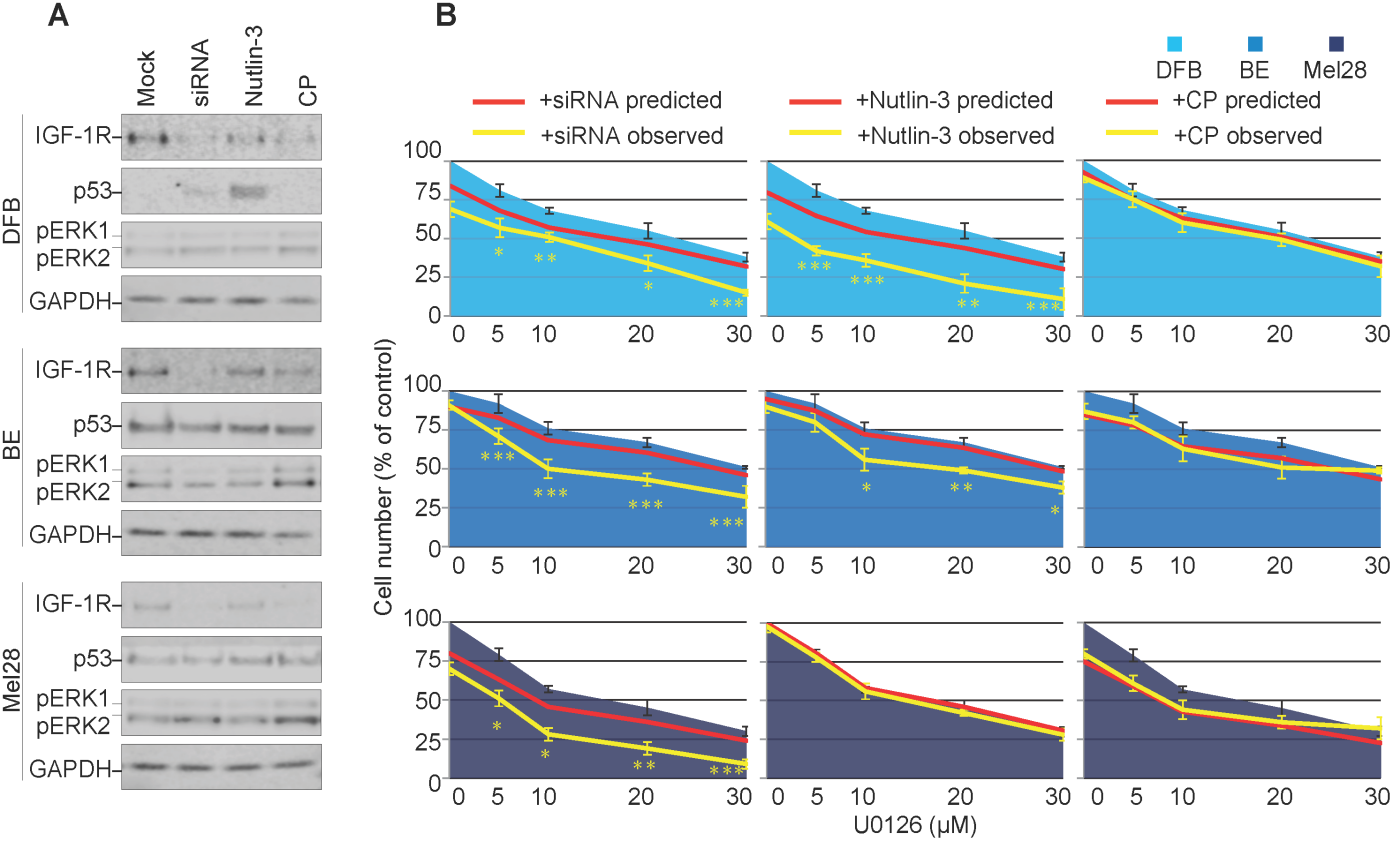
Effects of transient versus biased IGF-1R down-regulation on melanoma response to MEK1/2 inhibitor **(A)** DFB, BE and Mel28 melanoma cells were pre-treated with IGF-1R siRNA (48 h), 1 μM Nutlin-3 (12 h), 100 ng/ml CP (12 h) or mock (DMSO) treatment, followed by 24 h of 20 μM MEK1/2 inhibitor U0126 alone. Lysates were analyzed by WB for levels of IGF-1R, p53, phosphorylated (p) ERK1/2, and GAPDH as a loading control. **(B)** Parallel samples, receiving the same sequential treatment as in (A), were analyzed for total cell number by PrestoBlue fluorescence 48 h after MEK1/2 inhibition. Cell number after U0126 (single agent) treatment is shown as blue area. Predicted sensitivity of combined treatments, if additive, is displayed as red line. Observed cell sensitivity to combination treatments is shown as a yellow line. Data displayed as mean ± S.E.M from three independent experiments, expressed as % of mock treated controls. Statistical analysis: Cell number in observed combination treatments compared to predicted. *P<0.05, **P<0.01, ***P<0.001.

So far, our results demonstrate that three different strategies decreased the overall levels of the IGF-1R, however with different outcomes on the signaling characteristics of the down-regulated receptor. To directly investigate the effects of biased IGF-1R down-regulation on MEK1/2 inhibition, we evaluated the ability of melanoma cells to survive in the presence of MEK1/2 inhibitors (U0126). Briefly, biased or neutral, balanced IGF-1R down-regulation was induced by treating the melanoma cells grown in complete media, with Nutlin-3, CP or IGF-1R siRNA, the IGF-1R inhibitors were removed, and the cells treated with and without U0126. The effects of the combined treatment on receptor expression, signaling and p53 activation were verified by WB. All three regimes reduced IGF-1R in all cell lines, except for Nutlin-3 in Mel28 (Figure 4A). Wild type p53 cell line DFB exhibited an up-regulation of p53 upon Nutlin-3 treatment, whereas no other treatment in any cell line led to notable p53 changes (Figure 4A). More importantly, in all cell lines, the combination of siRNA or Nutlin-3 with U0126 lowered the level of pERK1/2. On the other hand, combination of CP with U0126 maintained an increased pERK1/2 level as compared to the untreated control, indicating a competition between CP-induced biased signaling and MEK1/2 inhibition (Figure 4A).

Parallel samples, receiving the same sequential treatment, were analyzed for total cell number 48 h after MEK1/2 inhibition (Figure 4B). At the end of the experiment, in the absence of MEK1/2-inhibitors, siRNA, Nutlin-3 or CP alone only slightly decreased the total number of cells, ranging from 5-20%, with CP being most effective in the Mel28 cell lines, and the Nutlin-3 effect more evident in the wild type p53 DFB (Figure 4B, red line point 0). To assess possible synergy, these single agent sensitivity data were combined with the MEK1/2 inhibitor alone dose-response curve, in identical conditions, calculated from a parallel experiment (Figure 4B, blue area), to produce a predicted dose-response curve, assuming only additive response of the two treatments (Figure 4B, red line), and compared with the observed response of drug combination (Figure 4B, yellow line). In the case of balanced IGF-1R down-regulation (siRNA), the cell sensitivity to MEK1/2 inhibitors increased in all cell lines, beyond what was predicted for an additive response, with a resultant IC50 of about half of the values obtained by MEK1/2 inhibition alone, illustrating a synergistic response to the combination treatment. A similar synergistic pattern was displayed by the DFB and BE cells in the case of Nutlin-3 treatment (the Nutlin-3 effect in DFB is stronger than siRNA) yet the response of Mel28 to MEK1/2 inhibitor was unchanged by Nutlin-3. In the case of CP-pre-treatment, the response to MEK1/2 inhibitors was additive, with the actual dose-response curve not significantly different from the predicted one.

These results confirm that IGF-1R down-regulation can improve response to MEK1/2 inhibition in melanoma cells, but the ultimate response differs depending on the signaling mechanisms activated alongside receptor internalization. A balanced or transiently biased approach (siRNA/Nutlin-3) synergizes with MEK1/2 inhibition, whereas a sustained biased agonist (IGF-1R targeting anybody) does not.

## DISCUSSION

**Figure 5:**
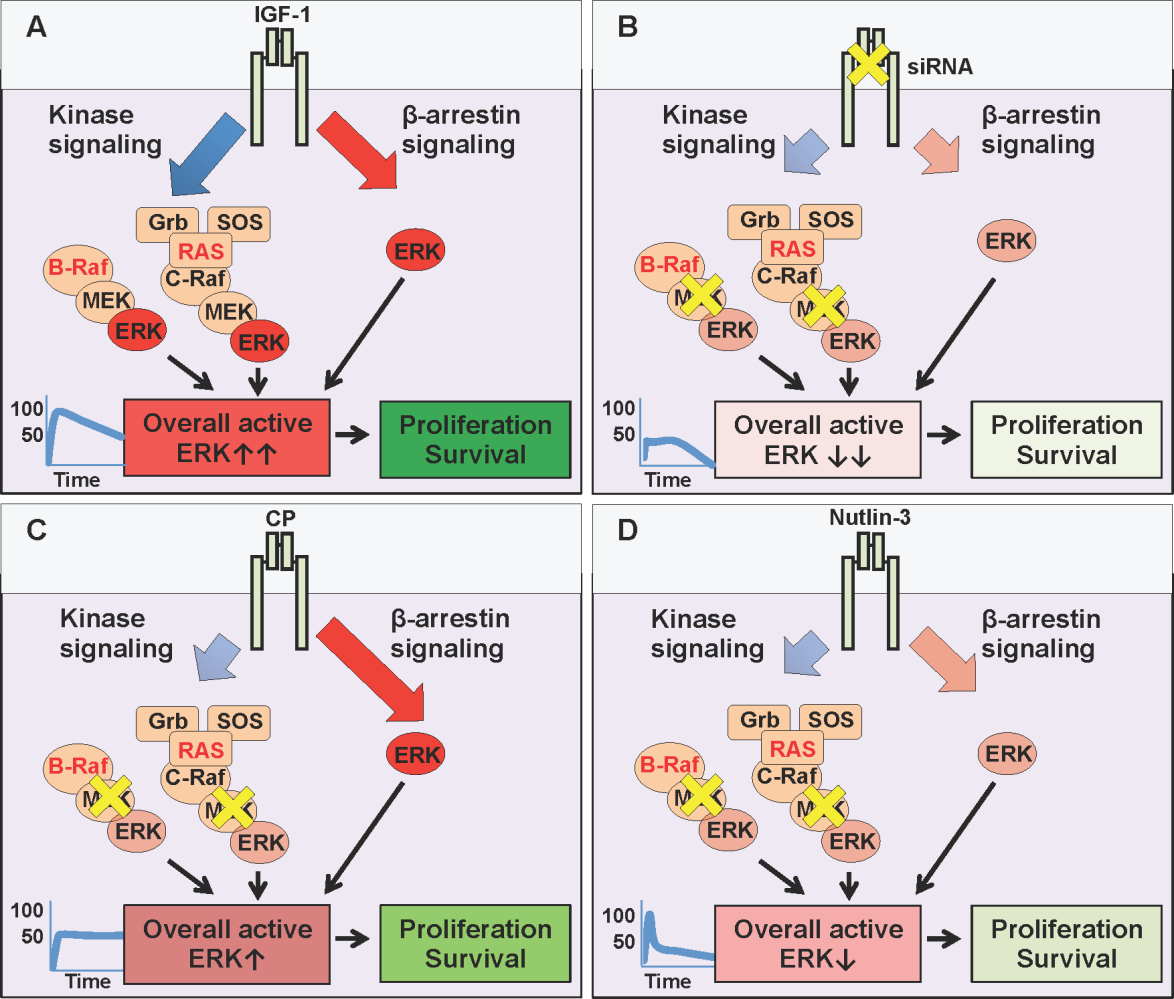
MEK and IGF-1R co-targeting **(A)** The natural (balanced) ligand, Insulin-like growth factor type 1 (IGF-1) binds to the IGF-1R and activates all downstream signaling pathways simultaneously: canonical kinase signaling (MAPK and PI3K/Akt (not shown)) and kinase-independent β-arrestin signalling (MAPK). In conditions with either BRAF or RAS mutations, the overall ERK activity is very high (graph, ***↑↑***) driving cellular survival and proliferation. **(B)** Targeting the IGF-1R with small interfering RNA (siRNA) prevents the translation of IGF-1R mRNA, thus reducing receptor levels at the cell surface (inhibition - yellow X), limiting all downstream pathways (kinase and β-arrestin). Small molecule U0126 further inhibits MEK, downstream of hyperactive RAS or BRAF. When combined, the resultant overall ERK activity is severely impaired (***↓↓***), decreasing cellular survival and proliferation. **(C)** Targeting the IGF-1R with specific antibody Figitumumab (CP-751871 (CP)) promotes receptor down-regulation, limiting its cell surface expression. However, this down-regulation triggers IGF-1R kinase-independent β-arrestin signaling (biased agonist). In co-targeting, this β-arrestin generated ERK activity competes with MEK inhibition (U0126), and resultant overall ERK activity remains high enough (***↑***),to sustain cellular survival and proliferation. **(D)** Targeting the IGF-1R with small molecule Nutlin-3 promotes receptor down-regulation, with transient β-arrestin signaling, insufficient to compensate for MEK inhibition. Reduced receptor expression combined with MEK inhibition, keeps overall ERK activity very low (***↓***) reducing cellular survival and proliferation.

The last few decades have witnessed a turning tide towards cancer targeted therapeutics and away from non-specific chemotherapeutic strategies. Whilst the greater-specificity and lessened-side-effect aims are mutually compatible, the main drawbacks are the resistance mechanisms associated with mono-therapy. Direct, specific targeting of one signaling module creates a selective pressure within an already unstable, hence adaptable genomic environment. MAPK inhibition, despite great initial clinical response, is rapidly associated with acquired resistance, including a switch in reliance to RTKs such as IGF-1R [5-7, 11, 13, 39]. In this context, our results confirm IGF-1R as a valid therapeutic target which supports anti-MAPK first-line treatment (Figure 5). We demonstrated that IGF-1 limits the effects of MEK1/2 inhibition in melanoma cells (Figure 5A), while siRNA, by preventing IGF-1R *de novo* synthesis with a proportional, balanced overall decrease of its signaling, greatly increases the efficacy of MAPK targeting (Figure 5B). These results agree with previous reports demonstrating that IGF-1R gene silencing improves melanoma response to various anti-cancer therapies [14, 17, 43]. However, we and others have shown that not all IGF-1R down-regulation strategies are equivalent in their ultimate effects [18, 25, 35, 44, 45]. Identification of biased signaling downstream of IGF-1R [20, 25, 27, 35, 46, 47] opened an intriguing possibility of controlling its signaling with more specificity and precision required for a multi-hit therapy. Hence, as a second key finding our study demonstrates that melanoma cell response to MEK1/2 inhibition with IGF-1R co-targeting critically depends on the biased or balanced conformation stabilized by the targeting agent. While IGF-1R siRNA proves the concept that co-targeting, by removing the “back up” resistance mechanism, both kinase and β-arrestin-dependent, (Figure 5B), enhances response to MEK1/2 inhibition, this approach does not currently represent a viable therapeutic intervention in human patients. One possible strategy would be the use of blocking antibodies, however our results indicate that an anti-IGF-1R antibody with sustained biased agonist properties (e.g. CP) does not synergize with MEK1/2 inhibitors, but instead competes with it, activating its own wave of MAPK signaling (Figure 5C). While highlighting the limited contribution of receptor down-regulation in enhancing the efficacy of MAPK targeting, our study reveals a novel and unexpected characteristic of this approach: CP acts to bias the receptor towards the endogenous, normally balanced ligand. Unbalanced signaling is generally considered a property of ligand-receptor interaction (e.g. either the ligand or the receptor are biased). In this case our interpretation is that pre-treatment of cells with CP stabilizes a biased receptor conformation that is preserved even when CP is removed and the natural ligand IGF-1 is available (Figure 5C). This long term MAPK enhancement may explain both the limited response to this single agent therapy, and its limited effects when combined with MEK1/2 inhibitors.

Small molecule inhibitor Nutlin-3 on the other hand, is a viable approach, as MAPK biased signaling associated with IGF-1R down-regulation is transient and not sufficient to protect cells in the longer term (Figure 5D).

Taken together, the co-targeting of MEK1/2 alongside IGF-1R inhibition with Nutlin-3 provides a powerful three-hit strategy. Firstly, MEK 1/2 inhibition targets the hyperactive MAPK (Figure 5D). Secondly, concomitant IGF-1R down-regulation removes all receptor-dependent back up survival pathways, and thirdly, treatment with Nutlin-3 increases the p53 protein level in cells retaining wild type p53, meaning that they are more susceptible to p53-activated cell death mechanisms. An important question that remains to be answered is whether in such a triple-hit strategy the Nutlin-3 effects are critically dependent on p53-rescue or via IGF-1R downregulation by the means of Mdm2 activation. It has been reported that wild type 53 melanoma cells demonstrate enhanced growth suppression in response to Nutlin-3, suggesting p53 status as a potential biomarker identifying tumors responsive to p53-reactivation therapy [48]. Yet, wild-type p53 is not an absolute requirement as several studies identified mutant p53 cells responsive to Nutlin-3 treatment, and it is likely that p53-independent mechanisms also modulate the Nutlin-3 response [41, 48–51]. The precise mechanisms orchestrating the response to Nutlin-3 are not fully recognized, however it is worth pointing out that Nutlin-3 not only stabilizes p53 but also triggers Mdm2 activity. As Mdm2 is a known ubiquitin ligase for several substrates other than p53, including IGF-1R, it is not surprising that Nutlin mediated Mdm2 activation alters diverse cellular functions such as cell proliferation, differentiation, migration and survival even in a p53- mutant or null background [35, 48–51]. A possible scenario, currently under investigation in our laboratories, aiming to explain this variability of cellular responses, is that Mdm2 acts as a hub adjusting the duration, magnitude and subcellular compartmentalization of signaling complexes, which are then interpreted by the cells for appropriate proliferative, death or migratory responses.

Taken together, our results advocate for the concept that IGF-1R targeting synergizes with MEK1/2 inhibition, by removing a crucial back-up pathway available to melanoma cells. The IGF-1R down-regulation strategy should be neutral or only transiently biased in nature. Small molecule Nutlin-3 offers such a strategy, and hence provides a possible therapeutic approach for further study.

## MATERIALS AND METHODS

### Cell lines and materials

SK-Mel28 (Mel28) cell line was purchased from ATCC (via LGC Standards, Middlesex, UK), and DFB and BE were obtained from Rolf Kiessling, CCK, KI, Stockholm, Sweden [52]. Mel28 was grown in Dulbecco’s modified Eagle’s medium (DMEM) supplemented with 10% (vol/vol) fetal bovine serum (FBS) and 1% penicillin/streptomycin (P/S). DFB and BE were grown in RPMI supplemented with 10% FBS and 1% P/S. DFB contains a homologous ^V600E^BRAF mutation on a wild type p53 background, SK-MEL28 (Mel28) contains the same homologous ^V600E^BRAF mutation but with a p53 mutation in codon 154, and BE contains a heterologous ^Q61R^NRAS mutation with a hot-spot mutation in the p53 codon 248 [41]. All cell lines were sequenced to verify their p53/RAS/RAF mutations [35, 52, 53], authenticated by short tandem repeat (STR) profiling (10/2016, Uppsala Genome Centre, Sweden), and tested regularly for mycoplasma contamination.

Nutlin-3 was dissolved in dimethyl sulphoxide (DMSO) at 2 mM and diluted in cell medium before use. IGF-1R targeting antibody CP 751871 (CP) was a kind gift from Pfizer (Pfizer, NY, USA). U0126 (1,4-diamino-2,3-dicyano-1,4-bis (2-aminophe-nylthio butadiene)) MEK 1/2 inhibitor was from Calbiochem (Nottingham, UK). Recombinant human insulin-like growth factor-1 (IGF-1) ligand was dissolved in 2% bovine serum albumin (BSA) at a concentration of 50 mg/mL. All materials were from Sigma Aldrich Ltd. (St Louis, MO, USA) unless otherwise stated.

### siRNA transfection

Cells were transfected with small interfering RNA (siRNA) against IGF-1R (Ambion, Life Technologies, s7211, 5’ GCAUGGUAGCCGAAGAUUUtt 3’) using reverse transfection (adding suspended cells to transfection mixture in cell culture vessels) and RNAiMAX (Life Technologies) according to manufacturer’s protocol.

### SDS-PAGE (sodium dodecyl sulphate-polyacrylamide gel electrophoresis) and western blot analysis

Cell lysates were prepared and subjected to western blot (WB) analysis as described in detail elsewhere [35]. Briefly, cells were lysed in LDS sample buffer and analyzed by SDS-PAGE on 4-12% Bis-Tris gels (Invitrogen, Carlsbad, CA, USA). Following electrophoresis, proteins were transferred to nitrocellulose membrane, blocked, and incubated overnight with appropriate primary antibody. Following washing, they were incubated with HRP-labeled secondary antibody from Pierce (Rockford, IL, USA). Detection was performed using enhanced chemiluminescent substrate (Pierce) and exposure to X-ray film or imaged using the Odyssey system (Li-cor, Lincoln, NE, USA). Primary antibodies diluted in 5% BSA in TBST for IGF-1R (1:2,000), phospho-ERK1/2 (1:2,000), total ERK1/2 (1:2,000) phospho-Akt (1:2,000) and phospho-IGF-1R (1:2,000) were from Cell Signaling Technologies (via BioNordika, Stockholm, Sweden). Primary antibodies diluted in 5% non-fat dry milk in TBST for p53 (sc-126, 1:1,000) and GAPDH (sc-25778, 1:4,000) were from Santa Cruz Biotechnologies (Santa Cruz, CA, USA).

### Densitometric analysis of western transfer analysis

Western blot analysis bands were quantified using the BioRad Quantity 1-D Analysis software (BioRad Laboratories AB, Stockholm, Sweden).

### Cell viability assay

Cells were plated at 10,000 cells/well in complete medium in 96 well tissue culture plates, and treated as described. Cell viability was measured by PrestoBlue cell viability assay (Invitrogen) according to the manufacturers’ protocols. For PrestoBlue, fluorescence was measured by excitation at 560 nm and detecting emission at 590 nm. Cell number was interpolated from standard curves of known cell number. The IC50 of the strategy was defined as the concentration required to reduce the number of cells to 50% of the untreated.

### Statistics

Where indicated, data from a minimum of three independent experimental replicates of two conditions were compared using a two-tailed, unpaired t-test assuming equal variance. Data expressed with error bars show mean ± standard error of the mean (SEM) from three independent biological experiments. A threshold value of P = 0.05 was chosen for testing any null hypothesis. Significance is given as * = P < 0.05, ** = P < 0.01, *** = P < 0.001.

## Abbreviations

MEK: MAPK/ERK kinase (Mitogen-activated protein kinase/Extracellular signal-regulated kinase kinase)
IGF-1R: insulin-like growth factor type 1 receptor
MAPK: mitogen-activated protein kinase
ERK: extracellular signal-regulated kinase
IGF-1: insulin-like growth factor type 1
RTK: receptor tyrosine kinase
GPCR: G-protein coupled receptor
siRNA: small interfering ribonucleic acid
mRNA: messenger ribonucleic acid
GAPDH: glyceraldehyde 3-phosphate dehydrogenase
IRS: insulin receptor substrate
PI3K: phosphoinositide 3-kinase
IC50: half maximal inhibitory concentration
CP: figitumumab (CP-751871)
FBS: fetal bovine serum
P/S: penicillin/streptomycin
STR: short tandem repeat
DMSO: dimethyl sulphoxide
U0126: 1,4-diamino-2,3-dicyano-1,4-bis (2-aminophe-nylthio butadiene)
BSA: bovine serum albumin
WB: western blot
SDS-PAGE: sodium dodecyl sulphate-polyacrylamide gel electrophoresis
SEM: standard error of the mean
TKIs: tyrosine kinase inhibitors
SFM: serum free media
